# ACR testing of a dedicated head SPECT unit

**DOI:** 10.1120/jacmp.v15i4.4632

**Published:** 2014-07-08

**Authors:** William F. Sensakovic, Matthew C. Hough, Elizabeth A. Kimbley

**Affiliations:** ^1^ Department of Radiology Florida Hospital Orlando FL; ^2^ College of Medicine Florida State University Orlando FL; ^3^ College of Medicine University of Central Florida Orlando FL; ^4^ Diagnostic Medical Physics/Radiation Safety Florida Hospital Orlando FL; ^5^ Department of Nuclear Medicine Florida Hospital Orlando FL USA

**Keywords:** image quality, SPECT, image perception

## Abstract

Physics testing necessary for program accreditation is rigorously defined by the ACR. This testing is easily applied to most conventional SPECT systems based on gamma camera technology. The inSPira HD is a dedicated head SPECT system based on a rotating dual clamshell design that acquires data in a dual‐spiral geometry. The unique geometry and configuration force alterations of the standard ACR physics testing protocol. Various tests, such as intrinsic planar uniformity and/or resolution, do not apply. The Data Spectrum Deluxe Phantom used for conventional SPECT testing cannot fit in the inSPira HD scanner bore, making (currently) unapproved use of the Small Deluxe SPECT Phantom necessary. Matrix size, collimator type, scanning time, reconstruction method, and attenuation correction were all varied from the typically prescribed ACR instructions. Visible spheres, sphere contrast, visible rod groups, uniformity, and root mean square (RMS) noise were measured. The acquired SPECT images surpassed the minimum ACR requirements for both spatial resolution (9.5 mm spheres resolved) and contrast (6.4 mm rod groups resolved). Sphere contrast was generally high. Integral uniformity was 4% and RMS noise was 1.7%. Noise appeared more correlated than in images from a conventional SPECT scanner. Attenuation‐corrected images produced from direct CT scanning of the phantom and a manufacturer supplied model of the phantom demonstrated negligible differences.

PACS numbers: 87.57.C‐, 87.57.uh, 87.63.lj

## I. INTRODUCTION

The Medicare Improvement for Patients and Providers Act (MIPPA) requires ongoing accreditation of single photon emission computed tomography (SPECT) and other advanced diagnostic imaging services, starting on January 1, 2012, in order for the provider to receive reimbursement for such services.^(1)^ The American College of Radiology (ACR) is a recognized accrediting body under MIPPA. In addition to other requirements, the ACR mandates each SPECT scanner pass minimum thresholds on specific image quality and equipment tests in order for a facility to receive nuclear medicine accreditation.^2^, ^3^, ^4^

The majority of SPECT systems commercially available and currently in use (including those from all major vendors) are comprised of one or more gamma camera detectors (with interchangeable collimators) that rotate about the patient, acquiring projection images to form a sinogram that is reconstructed into the final dataset. Although there are some variations regarding filters, number of gamma camera detectors, total angular degrees of rotation, scintillator material, filters, collimators, and reconstruction algorithms, most SPECT systems share a very similar scan geometry and hardware components. The specific testing methodology prescribed by the ACR (the standard ACR protocol) was created based on these conventional systems.

The inSPira HD (Neurologica, Danvers, MA) is a dedicated head SPECT system that differs substantially from conventional SPECT scanners in both scanning geometry and hardware configuration. These differences force deviations from the standard ACR protocol during physics testing. The purpose of this study is to present our experience testing the inSPira HD for ACR accreditation, emphasizing specific difficulties and areas where the testing methodology differs substantially from the standard ACR protocol. ACR testing results and results of several quantitative tests performed using the images acquired for ACR testing are also reported.

## II. MATERIALS AND METHODS

### A. Geometry and configuration of the inSPira HD

Conventional SPECT systems usually consist of one or more gamma camera detectors with interchangeable collimators. The gamma camera detectors are positioned an appropriate distance from the patient surface and then rotate about the patient to acquire images at different angles. These images are then used to create a sinogram that is reconstructed using filtered backprojection or iterative methods. Systems that are not coupled to a computed tomography (CT) scanner typically use Chang's multiplicative (or similar) method, with a fixed attenuation coefficient to ensure attenuation‐corrected images exhibit uniformity of a given activity concentration throughout the transaxial sections.^(5)^

**Figure 1:**
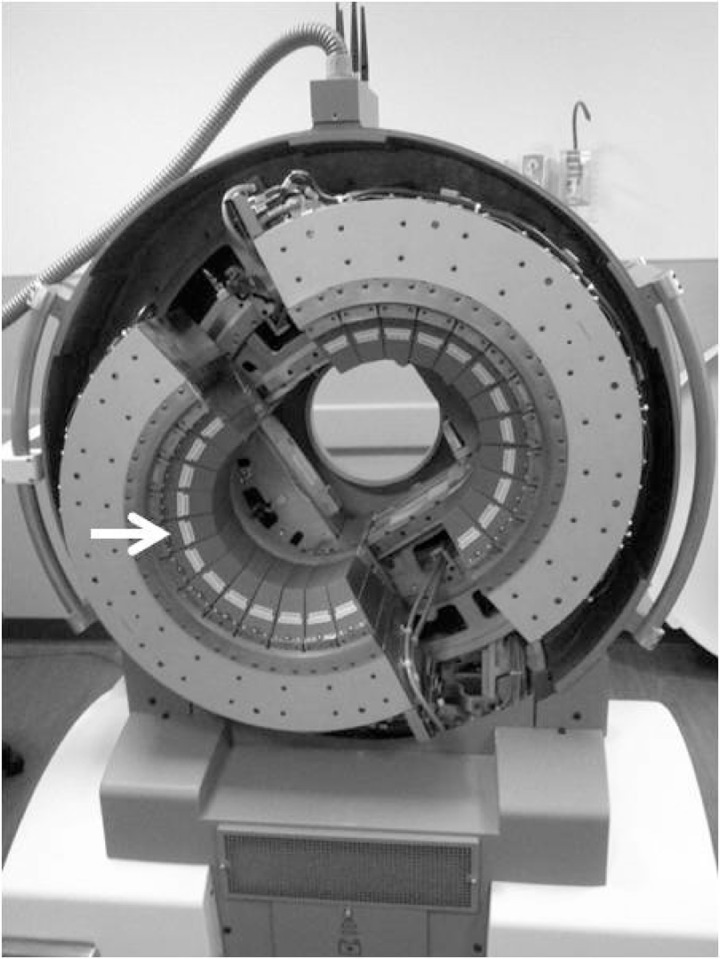
Neurologica inSPira HD showing photomultiplier tubes with scintillation crystals and “cone” collimators (white arrow) arranged three‐deep and in a semicircle on each clamshell. The clamshells spin and separate to acquire each image slice.

**Figure 2:**
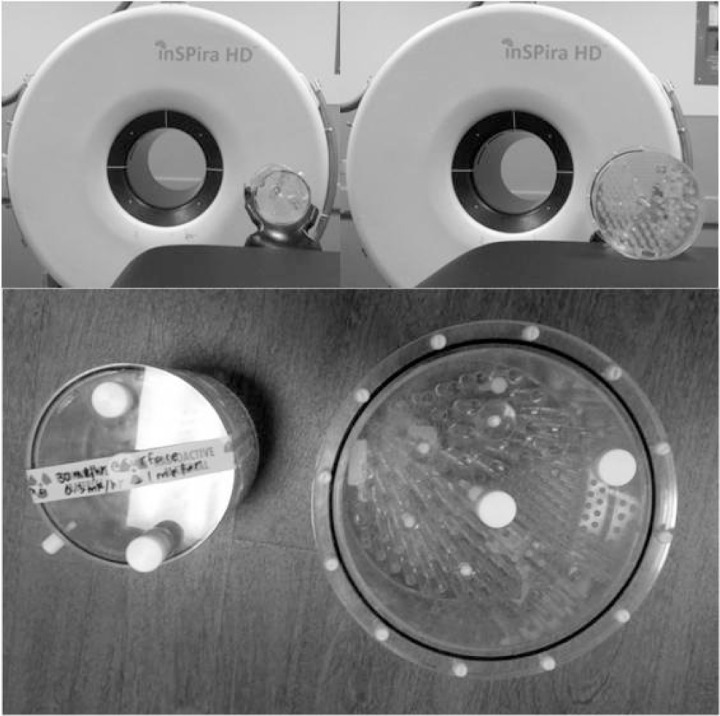
Scanner bore and phantom. Top: Small (left) and flanged Deluxe (right) Phantoms next to SPECT scanner bore (note that the flanged Deluxe Phantom cannot fit in the head holder and cannot fit in the bore while resting on a platform). Bottom: Small (left) and flanged Deluxe (right) Phantoms.

In contrast to the conventional SPECT system, the Neurologica inSPira HD consists of two clamshells (Fig. 1) creating a 29 cm diameter bore similar to a small CT scanner (Fig. 2). Each clamshell contains 36 photomultiplier tubes (PMTs) arranged in groups of three PMTs axially.

**Figure 3:**
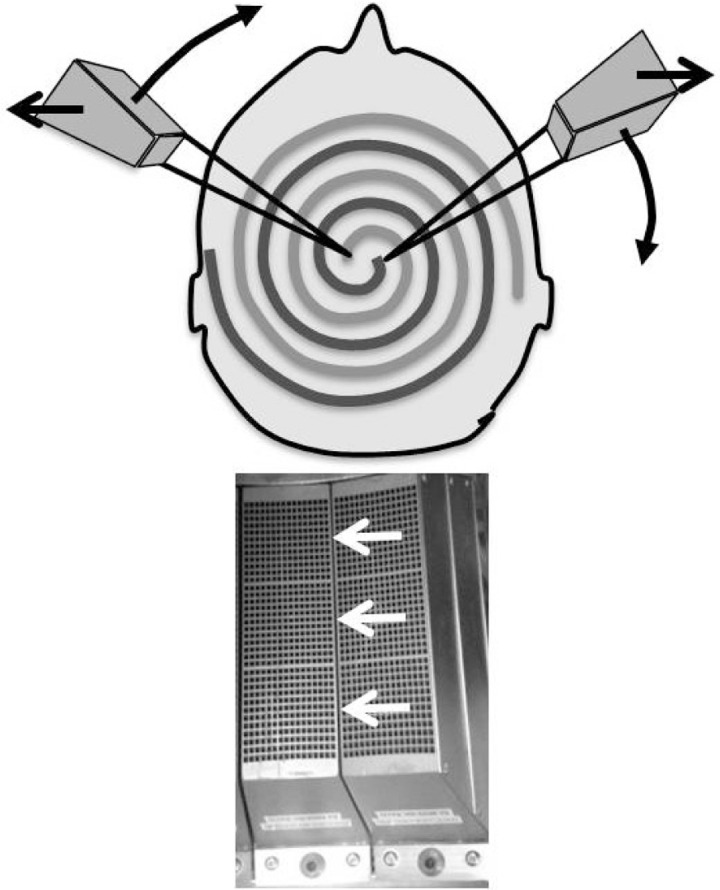
Cone collimator and acquisition geometry. Bottom: A fixed cone collimator is attached to the front of each photomultiplier tube (PMT) assembly. PMTs are stacked 3‐deep along the axial direction of the scanner (white arrows). Top: Two PMTs on opposing sides of the clamshell. During acquisition the clamshell spin and separates (black arrows indicate motion) causing the fixed focus of each clamshell to sample image space in a spiral.

A fixed focused “cone” collimator is attached to each PMT, and all cones are focused on a common point (center of the bore when clamshell is closed). For each section to be acquired, the clamshell spins multiple times around the patient as the clamshell opens. This causes the scanner to sample the image space in a dual‐spiral geometry (Fig. 3). A proprietary iterative reconstruction algorithm is applied to reconstruct the data into transaxial images. The reconstruction algorithm is an iterative digital tomosynthesis algorithm, utilizing precise numerical models of the collimators and detector system. There is no subsetting of the data (as is present in the popular ordered‐subset expectation maximization algorithm); all information is used in the reconstruction. The reconstruction normally is set to run for 60 iterations. If attenuation correction is selected, then the reconstruction algorithm incorporates a detailed attenuation model derived from a CT scan during reconstruction. The CT may be the patient's scan or one of several preloaded model scans available on the system (e.g., ACR small phantom, adult head, pediatric head three‐month‐old). CT scans are manually aligned to the SPECT scan by the technologist using a rigid registration method. No other postreconstruction processing (e.g., edge‐enhancement or Chang correction) is applied (M. Dickman of Neurologica, personal communication, October 15, 2013).

### B. Review of recommended setup and required testing

The ACR prescribes a specific physics testing methodology for phantom images to quantify SPECT image quality for accreditation (standard ACR protocol).^2^, ^3^, ^4^ The ACR further states that specific phantoms are to be used for SPECT testing. The conventional SPECT system will use the Deluxe Phantom (Data Spectrum Corp. Durham, NC), which has a 20.4 cm internal diameter. The ACR allows the use of the 14 cm internal diameter Small SPECT Phantom (Data Spectrum Corp. Durham, NC), referred to as “Small Phantom” in this study, for a limited subset of approved cameras. The inSPira HD is not currently included in this list.^(3)^ Recommended activity is between 5 and 15 mCi (185‐555 MBq) for the Small Phantom, so that total acquired counts are ∼20 million million.

ACR testing for a conventional SPECT system is broken down into planar and SPECT components. Planar testing components include use of Tc‐99m or Co‐57 for either intrinsic or system flood images, for planar field uniformity of each gamma camera detector, and for planar spatial resolution using the rod section of the phantom. Quality control (QC) information related to the most recent center of rotation and flood uniformity tests are also recorded. For SPECT testing, the phantom is aligned lengthwise along the scanner axis and centered. The uniform portion of the phantom is placed “head first” into the scanner, and the spheres are oriented such that they are increasing with size clockwise from the top. The highest resolution low‐energy collimator typically used in clinical practice should be used for SPECT testing. Acquisitions should be 120 or 128 views over 360° (or clinically used angular sampling if gamma camera detectors do not rotate). Gamma camera detectors should be placed as close as possible to rotation center. Images should be acquired with a matrix of 128×128 pixels with pixel spacing between 2.7 mm and 3.3 mm (zoom can be used to satisfy this criterion). Slices should be 6‐9 mm thick, and adjacent sections may be summed to attain this level of thickness.

These images are used to assess uniformity (in the uniformity section of the phantom) and cold contrast (using the spheres positioned in the phantom). The uniformity section should be absent of any obvious artifacts for satisfactory passing of ACR testing. In the contrast (sphere) section of the phantom, the 19.1 mm sphere must be visible with low contrast for satisfactory passing of ACR testing.

A single image summing five adjacent images through the rod section is submitted to the ACR for review to assess system resolution. Seventy‐five percent of the 11.1 mm rods must be visible with low contrast in the rod group for satisfactory passing.

Images may use any reconstruction technique during ACR testing, as long as all user‐controlled parameters are listed. The ACR gives recommendations if filtered backprojection is applied due to its widespread use during ACR testing. Specifically, a Butterworth filter with slope 6 and cutoff 0.55 should be applied (though this is not required). Finally, Chang's multiplicative method with a specified coefficient (∼0.12 1/cm) is often applied to create attenuation‐corrected images for non‐SPECT/CT systems.^(5)^

### C. Testing methodology for the inSPira HD

The Small Phantom with an activity of 14.7 mCi (543.9 MBq), at the upper end of the ACR suggested range, was used in all ACR and quantitative testing performed for this study. Unlike conventional SPECT systems, centering and aligning the phantom in the scanner bore is facilitated by the presence of a crosshair laser inside the bore (similar to that found in a typical CT system). Phantom positioning was as otherwise described by the standard ACR protocol. Small Phantom images were acquired using 60 s per transaxial section, slice thickness of 3.125 mm, and an 81×81 matrix, which produced a pixel spacing of 3.125 mm. A CT scan of the Small Phantom was supplied to the manufacturer's proprietary reconstruction algorithm to generate attenuation‐corrected images. No postprocessing filter was explicitly added by the operator.

The unique design of the inSPira HD limited what standard ACR tests could be performed. Modifications necessary to the standard ACR protocol due to the unique geometry of the inSPira HD are detailed in the Results and Discussion section below. ACR tests for cold contrast (number of spheres visible to an observer), resolution (number of rod groups visible to an observer), and qualitative evaluation of uniformity/artifact/noise were performed for both attenuation‐corrected and nonattenuation‐corrected images. Image assessment was independently performed by two medical physicists. The smallest visible sphere and smallest visible rod group were reported, as well as qualitative descriptions of noise and uniformity.

Three quantitative tests that were not ACR requirements were also performed, using the attenuation‐corrected images created for ACR testing. A quantitative measure of uniformity (integral uniformity) was calculated^(6)^ using the uniformity section of the Small Phantom and the equation:

(1) $$\text{Integral Uniformity(\%)=100*}\frac{pix\_count_{\text{max}}-pix\_count_{\text{min}}}{pix\_count_{\text{max}}-pix\_count_{\text{min}}}$$

where $pix\_count_{max}$ and $pix\_count_{min}$ are the maximum and minimum counts for a pixel in the central 15×15 pixel area of the uniform section of the phantom. A quantitative measure of cold contrast was calculated^(6)^ using the spheres in the Small Phantom and the equation:

(2) $$\text{Contrast(\%)=100*}\frac{pix\_count_{Uniform\;Avg.}-pix\_count_{Sphere\;Min}}{pix\_count_{Uniform\;Avg}}$$

where $pix\_count_{Uniform\;Avg}$. is the average count of all pixels from the uniform region of the phantom, and $pix\_count_{Sphere\;Min}$. is the minimum count from any pixel in the sphere. Finally, root mean square (RMS) noise was calculated^(6)^ using the uniformity section of the Small Phantom and the equation:

(3) $$\text{RMS}\;\text{noise(\%)=100*}\frac{pix\_count_{StDev.}}{pix\_count_{Avg.}}$$

where $pix\_count_{St\;Dev}$. is the standard deviation of the counts in the central 15×15 pixels from the uniform region of the phantom, and $pix\_count_{Avg}$. is the average of the counts in the central 15×15 pixel area of the uniform section of the phantom.

Attenuation correction can be initialized for the inSPira HD using either a user‐provided CT scan or a manufacturer‐provided preloaded model. Attenuation‐corrected images derived from each of the two different initializations were qualitatively compared. Finally, the Small Phantom was scanned at seven different activities (range: 0.21 mCi (7.77 MBq) to 20.4 mCi (754.8 MBq)) with the same acquisition parameters used for ACR and quantitative testing. The machine‐reported total number of counts was recorded for each injected activity. A line was fit to the graph of machine‐reported counts versus phantom activity, and an equation for the fitted line was used to determine the Small Phantom activity necessary to produce the 20 million counts recommended by the ACR for testing SPECT systems using the Small Phantom.

## III. RESULTS & DISCUSSION

Our inSPira HD SPECT scanner received ACR accreditation in 2013. It is believed that this is the first instance of ACR accreditation for the inSPira HD system. We believe that this validates the modified ACR testing methods detailed in this study, and confirms that they are suitable to use as a template when testing the inSPira HD (or similar system). The unique geometry of the inSPira HD forces modifications to the standard ACR testing procedure. Due to the 29 cm bore size (Fig. 2), it is not possible to fit the Deluxe Phantom into the scanner when it is placed on the patient headrest. Further, the 20 cm FOV of the scanner implies that the entire diameter of the Deluxe Phantom could not be visualized by the inSPira HD. Thus, all ACR testing (as well as any related QC testing) must instead use the Small Phantom, though the inSPira HD is not currently included in the ACR list of scanners with exemptions to use the Small Phantom for accreditation testing. The activity used for our accreditation testing was just below the highest ACR recommended activity for the Small Phantom (15m Ci or 555 MBq). Although our ACR testing (based on standard ACR protocol) was performed at this high activity level, subsequent phantom studies produced a phantom activity‐to‐total counts relationship (using the Small Phantom and acquiring for 60 s/image) of:

(4) $$\text{Total Counts}=7.176\times 106^{*}\text{Activity in mCi}=1.939\times 105^{*}\text{Activity in MBq}$$

with an $R^{2}$ value of 0.999 for the linear fit. Given this result, future testing using the Small Phantom in the inSPira HD could be performed at 3–4 mCi (111–148 MBq). This would ensure that the ACR‐recommended ∼20 million counts are achieved, while reducing the radiation exposure for the technologist and physicist; however, this is below the lowest ACR‐recommended activity for the Small Phantom (5 mCi or 185 MBq).

The inSPira HD does not support planar imaging (SPECT only), and thus tests related to planar imaging are not applicable to this system. The following tests were reported to the ACR as not applicable: uniformity, spatial resolution, system resolution, sensitivity, and maximum count rate. Neurologica recommends daily quality assurance checks for the inSPira HD using provided system software. After a source of user‐specified activity is placed in the scanner bore, software performs internal checks for offset, background noise, detector and energy window efficiency, encoder verification, and image verification. The software reports only pass or fail for each test and specifics of the testing methodology are not provided. The ACR submission for the inSPira HD reported that energy resolution and peaking met the manufacturer's specifications based on daily quality control records. Neither center‐of‐rotation nor high‐count flood uniformity is performed on the inSPira HD for SPECT imaging; so related quality control information was not submitted.

**Figure 4:**
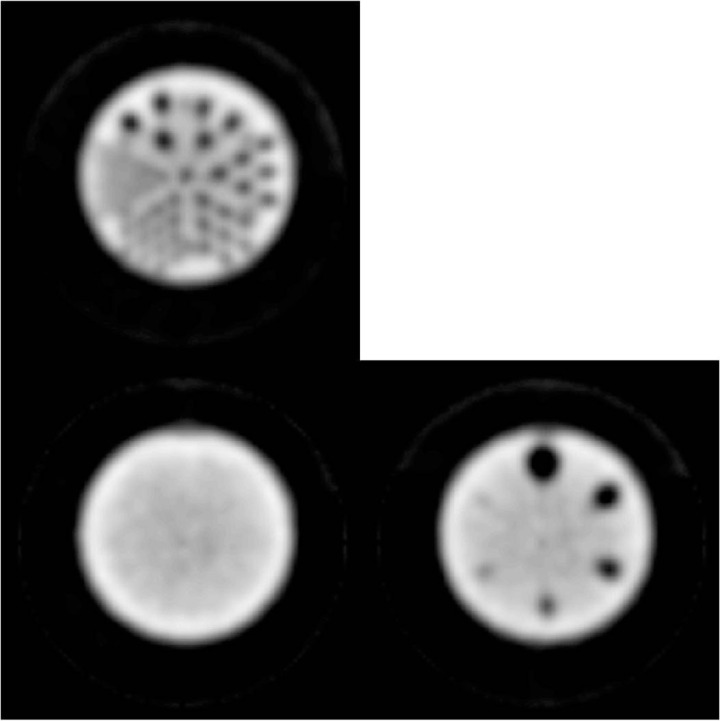
ACR phantom images without attenuation correction: Rod image (top) from summation of five sections demonstrating high SPECT resolution; uniformity image (bottom left) demonstrating bright ring due to lack of attenuation correction and coarse noise likely due to reconstruction algorithm; sphere image (bottom right) demonstrating excellent cold contrast even without attenuation correction.

Several acquisition parameters were modified from the standard ACR protocol due to the unique geometry of the inSPira HD. Acquisitions of the Small Phantom were performed using the cone collimator, as these are fixed and the collimator cannot be changed. Similarly, the distance between the detectors and the surface is not controlled by the operator. The focal distance of the collimator is fixed at 15 cm and the dual‐spiral acquisition ensures that each point in the phantom is 15 cm from the detector during acquisition. In contrast, the standard ACR protocol recommends a 20 cm gamma camera radius of rotation; however, points deeper in the phantom will actually be further from the detector during acquisition. The specific number of views is not operator‐controlled and, due to the scanning geometry (a dual spiral), the detectors actually revolve around the patient many times (∼30 revolutions per minute and 30 to 60 s scan time per transaxial section). Examination of the DICOM header in phantom images demonstrated a machine‐reported 168 frames with an angular increment of 2.14° for both 30 s and 60 s per slice acquisition times. Although a widely used reconstruction method during standard ACR testing of SPECT systems, filtered backprojection is not an option for reconstructing images in the inSPira HD. The reconstruction was performed using the proprietary iterative reconstruction algorithm provided by the manufacturer (no user controlled parameters exist to report). Reconstruction times for the Small Phantom (60 sections) were 120 min and 60 min with and without attenuation correction, respectively. Example images created by summing two adjacent slices (6.25 mm thickness) for the uniformity and sphere phantom sections and five adjacent slices (15.625 mm thickness) for the phantom rod section (per standard ACR protocol) are shown in Fig. 4.

**Figure 5:**
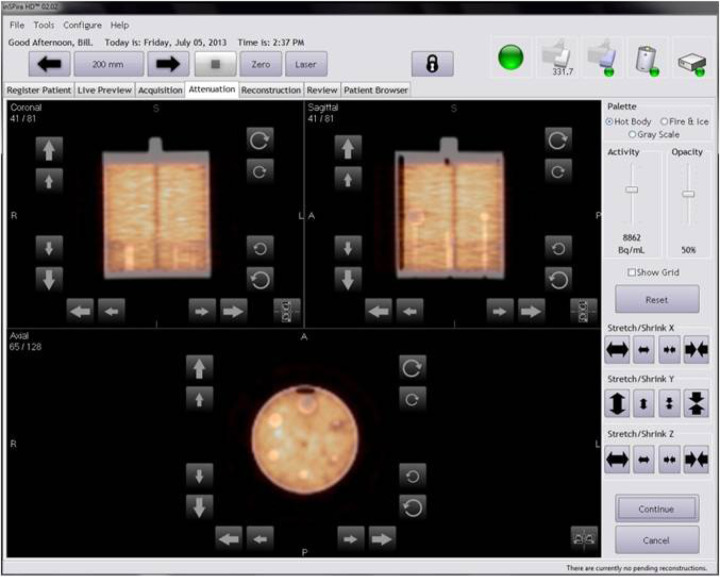
The interface for registering SPECT and CT images. Software allows for translation, rotation, scaling, and flipping about all three axes. The color lookup table (i.e., temperature map) for the SPECT scan and the blending between CT and SPECT data can also be altered to improve visualization of the alignment.

**Figure 6:**
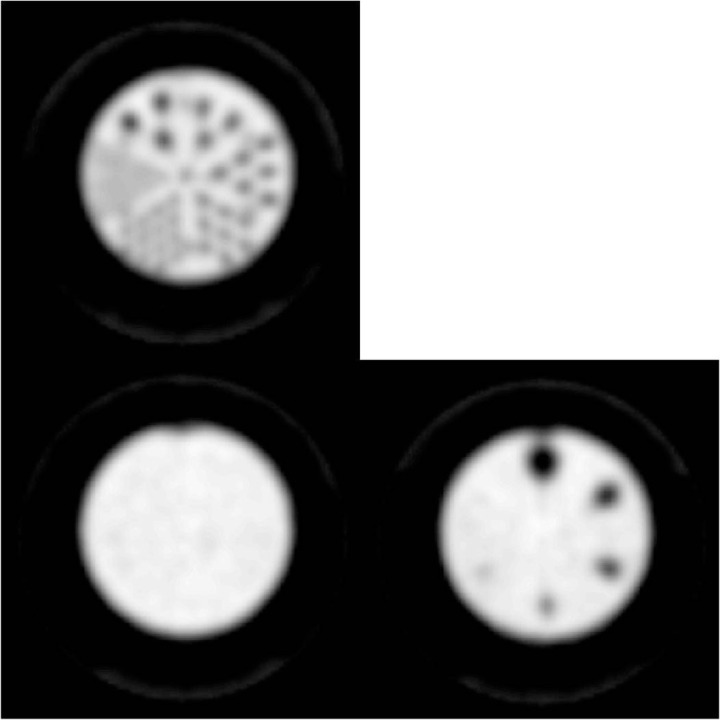
ACR phantom images with attenuation correction: Rod image (top) from summation of five sections demonstrating high SPECT resolution; uniformity image (bottom left); sphere image (bottom right).

**Figure 7:**
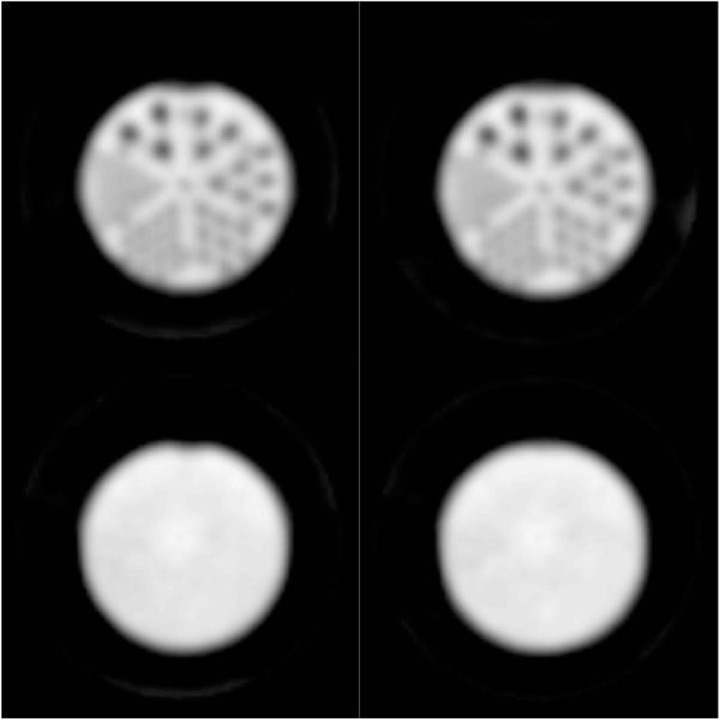
Comparison of attenuation correction methods. Left: Rod (top) and uniform (bottom) sections of the phantom with attenuation derived from a CT scan of the phantom. Right: Rod (top) and uniform (bottom) sections of the phantom with attenuation derived from a model of the Small Phantom that is preloaded in the scanner software.

ACT scan of the Small Phantom was supplied to the manufacturer's proprietary reconstruction algorithm to generate an attenuation‐corrected reconstructed dataset. Chang's (or similar) method for attenuation correction is not an option on the inSPira HD system. A single attenuation coefficient could not be reported to the ACR (as is standard for ACR testing) because the entire attenuation map is used in attenuation correction by the inSPira HD, as opposed to Chang's multiplicative (or a similar) method. Attenuation correction in the inSPira HD requires manual alignment of the CT and SPECT images using buttons which allow for translation, reflection, and rotations in predefined steps about each axis (Fig. 5). Images through the entire phantom and the summed image through rod sections with attenuation correction based on our acquired CT scan are shown in Fig. 6. The proprietary algorithm produced qualitatively uniform images without obvious artifact or overcorrection. Although we used CT images of the Small Phantom taken at our facility for ACR testing, attenuation‐corrected images derived using the template loaded in the scanner were not qualitatively different (Fig. 7).

All images produced by the inSPira HD were of subjectively high quality. Based on the independent evaluations of two medical physicists, rods were resolved at 6.4 mm (five groups) with low contrast, and spheres were visible at 9.5 mm (five spheres) with low contrast on images without attenuation correction. Attenuation correction qualitatively appeared to improve contrast uniformity; however, rods were still resolved at 6.4 mm (five groups) with low contrast, and spheres were still visible at 9.5 mm (five spheres) with low contrast on attenuation‐corrected images. Quantitative contrast for the five visible spheres (from smallest to largest) with attenuation correction was 24%, 50%, 73%, 89%, and 100%. Noise appeared more correlated than in images produced by a conventional SPECT scanner, and this is likely due to a combination of the high resolution of the inSPira HD and the proprietary reconstruction algorithm. RMS noise was 1.7% and integral uniformity was 4.0% for attenuation‐corrected images.

Future work will focus on direct image quality comparisons with a conventional SPECT scanner working under similar acquisition protocols and on more advanced image quality characterization (e.g., modulation transfer function, noise power spectrum) of the inSPira HD system.

## IV. CONCLUSIONS

The standard ACR protocol for image quality testing follows a prescribed recipe that its well for conventional gamma camera detector‐based SPECT systems. Newer anatomy‐targeted SPECT systems, which have unique scanning geometries and hardware configurations, require modifications to the typical testing setup and reporting. This study focused on the ACR testing of one such system — the Neurologica inSPira HD. It is believed that this is the first accreditation of the inSPira HD by the ACR. A description of procedures performed and results of our testing were reported and may be of interest to other institutions that will seek accreditation with the inSPira HD or a future SPECT system with similar scanning geometry and hardware coniguration.
